# Cigarette smoke regulates VEGFR2-mediated survival signaling in rat lungs

**DOI:** 10.1186/1476-9255-7-11

**Published:** 2010-02-13

**Authors:** John A Marwick, Indika Edirisinghe, Gnanapragasam Arunachalam, Christopher S Stevenson, William MacNee, Paul A Kirkham, Irfan Rahman

**Affiliations:** 1 National Heart and Lung Institute, Imperial College London, UK; 2 Respiratory Disease Area, Novartis Institute for Biomedical Research, Horsham, UK; 3 Edinburgh Lung and the Environment Group Initiative Colt Laboratories, MRC Centre for Inflammation Research, University of Edinburgh, Edinburgh, UK; 4 Department of Environmental Medicine, Lung Biology and Disease Program, University of Rochester Medical Centre, Rochester, NY, USA

## Abstract

**Background:**

Vascular endothelial growth factor (VEGF) and VEGF receptor 2 (VEGFR2)-mediated survival signaling is critical to endothelial cell survival, maintenance of the vasculature and alveolar structure and regeneration of lung tissue. Reduced VEGF and VEGFR2 expression in emphysematous lungs has been linked to increased endothelial cell death and vascular regression. Previously, we have shown that CS down-regulated the VEGFR2 and its downstream signaling in mouse lungs. However, the VEGFR2-mediated survival signaling in response to oxidants/cigarette smoke (CS) is not known. We hypothesized that CS exposure leads to disruption of VEGFR2-mediated endothelial survival signaling in rat lungs.

**Methods:**

Adult male Sprague-Dawley rats were exposed CS for 3 days, 8 weeks and 6 months to investigate the effect of CS on VEGFR2-mediated survival signaling by measuring the Akt/PI3-kinase/eNOS downstream signaling in rat lungs.

**Results and Discussion:**

We show that CS disrupts VEGFR2/PI3-kinase association leading to decreased Akt and eNOS phosphorylation. This may further alter the phosphorylation of the pro-apoptotic protein Bad and increase the Bad/Bcl-xl association. However, this was not associated with a significant lung cell death as evidenced by active caspase-3 levels. These data suggest that although CS altered the VEGFR2-mediated survival signaling in the rat lungs, but it was not sufficient to cause lung cell death.

**Conclusion:**

The rat lungs exposed to CS in acute, sub-chronic and chronic levels may be representative of smokers where survival signaling is altered but was not associated with lung cell death whereas emphysema is known to be associated with lung cell apoptosis.

## Introduction

Maintenance of the microvasculature in the lung is critical for gas exchange, the integrity of the alveolar structure and tissue repair [1]. Cigarette smoke (CS)-induced emphysema is characterized by enlargement of the airspaces and a loss of alveolar structure [2,3]. Endothelial cell death and the regression of lung parenchyma, capillary density seen in emphysema may be linked to this loss of the alveolar structure [4,5].

Vascular endothelial growth factor (VEGF) plays vital role in development and maintenance of vasculature and tissue regeneration [6]. VEGF signaling on endothelial cells is involved in several key processes during wound healing including degradation of the extracellular matrix of existing vessels, migration and proliferation of capillary endothelial cells, formation of new capillaries and restitution of the air-blood barrier in the alveoli [1,7]. Targeted disruption of VEGF gene in mice impairs blood vessel formation, growth retardation and premature death [8]. Furthermore, deletion or inhibition of VEGF in specific tissues in adult mice has shown noticeable effects, mainly significant reduction in capillary density with tissue cell apoptosis [9].

VEGF signaling through VEGF receptor 2 or kinase insert domain receptor (a type III receptor tyrosine kinase) or protein-tyrosine kinase receptor FLk-1 (VEGFR2) is key in endothelial survival and the maintenance of the vasculature [10,11]).). VEGFR2 inhibition leading to endothelial cell death has been linked to both lung vascular regression and alterations in alveolar structure [12,13]) VEGF/VEGFR2-mediated endothelial survival signals is predominantly mediated through phosphatidylinositol-3-OH kinase (PI-3K) and its downstream target of the serine-theronine kinase Akt [10]. Akt is a general mediator of growth factor-induced survival and has shown to suppress the apoptotic death *in vitro *induced by a variety of stimuli, including growth factor withdrawal, cell-cycle discordance, loss of cell adhesion and DNA damage [14-17]. VEGF-mediated survival signaling is mediated through the upregulation of anti-apoptotic proteins such as Bcl-2 and A1 [18], and IAP (inhibitors of apoptosis proteins), survivin and IXAP (X-chromosome-linked IAP) [19], which may inhibit upstream caspases and terminal effecter caspases respectively.

Bad is an pro-apoptotic member of the Bcl-2 family proteins that can displace Bax binding to Bcl-2 and Bcl-xl, results in cell death [20,21]. Survival factor IL-3 can inhibit the apoptotic activity of Bad by activating intracellular signaling pathways that results in phosphorylation of Bad (Ser112 and Ser136) [22]. This further leads to binding of Bad to 14-3-3 proteins and inhibition of Bad binding to Bcl-2 and Bcl-xl [22]. Akt has been shown to promote cell survival via its ability to phosphorylate Bad at Ser136 residue [23].

VEGF and VEGFR2-mediated downstream signaling activates eNOS [24], and release nitric oxide (NO) [25]. The mechanism of cell survival by NO can be directly linked to increased neovascularisation and cell migration [26] or by increasing Bcl-2 expression [27]. Previously, we have demonstrated that CS-induced oxidative stress impairs VEGF-mediated VEGFR2 phosphorylation and VEGFR2 expression in both endothelial cells and mouse lung [28], and in emphysematous lungs of both smokers and non-smokers [4,29]. However, the mechanism of CS-induced VEGFR2-mediated impaired Akt and its downstream signaling leading to apoptotic cell death in lung has not been studied. Therefore, we hypothesized that CS regulates VEGFR2-mediated survival signaling via Akt-dependent pathways in rat lung. To test this hypothesis, rats were exposed to CS for different time points (3 days, 8 weeks and 6 months) and VEGFR2/PI3-kinase association, Akt, eNOS, Bad phosphorylation and active caspase levels were determined.

## Materials and methods

### Animals

Adult male Sprague-Dawley rats (323 ± 2.5 g) (Charles River, Margate, UK) were divided into 6 exposure groups: (a) 3 day sham exposed (n = 6), (b) acute 3 day CS exposed (n = 6), (c) 8 week sham exposed (n = 6), (d) sub-chronic 8 week CS exposed (n = 6), (e) 6 months sham exposed (n = 6) and (f) chronic 6 months CS exposed (n = 6). The rats were exposed to whole body CS generated from 2R4F research cigarettes (University of Kentucky, Lexington, Kentucky, USA, total particulate matter (TPM) concentration 27.1 ± 0.8 mg per cigarette) in 7 L smoking chambers at 4 cigarettes per day, Monday to Friday. To ensure a consistent exposure across exposed animals, cotinine levels were measured. Plasma cotinine levels were 2.66 ± 0.12 μM after 1 hr exposure (cotinine was not detectable in the plasma from air-exposed animals) and 0.51 ± 0.07 μM after 24 hr. There was no progressive increase in cotinine levels over 1 week of exposures. Carboxyhemoglobin level was measured immediately after the animals were removed from the chambers. A peak level of 42 ± 4.0 μM was reached after the 4^th ^cigarette, which quickly decreases after the exposure was stopped. Sham exposed animals where exposed to medical grade air under the same conditions as CS exposed animals. Rats were sacrificed 2 hr post-last exposure by intra-peritoneal injection of 200 mg sodium pentobarbital.

### Tissue processing

The lungs were excised from rats, the right lobe tied off and then snap frozen in liquid nitrogen for immunoblotting and immunoprecipitation experiments. The left lobe was inflated with 5 ml of 10% neutral buffered formalin and then immersed in NBF to complete fixation for 24 hr. The left lobe was then sliced tangentially into 6 slices, which were processed as two tissue blocks. Sections (3 μm) of the 4 central lung slices were cut using a Leica rotary microtome. The sections were mounted on to Polysine slides (Surgipath Europe Ltd, Cambridge, UK) and dried overnight at 37°C.

### Immunohistochemistry

Briefly, lung sections were dewaxed in xylene, rehydrated and endogenous peroxidase inhibited with 0.5% hydrogen peroxide in methanol for 10 minutes. Sections were stained with anti-active caspase 3 (Abcam, Cambridge, UK), overnight at 4°C. Immunodetection was preformed using biotinylated rabbit anti-mouse IgG antibody/reagent (Dako Cytomation, Cambridgeshire, UK), SABC reagent (Dako Cytomation, Cambridgeshire, UK), and 3,3'-diaminobenzidine (DAB) (Sigma, Dorset, UK). The nuclei were counterstained with Cole's haematoxylin solution. Tonsil was used as a positive control and for negative controls the primary antibody was omitted from one section of each of the samples. Two fields to the right of the large airway in two pieces of the left lobe were counted (i.e. 4 fields, total area approximately 6.5 mm^2^). When there was a difference of more than 5 cells/mm^2 ^between the average counts of 2 and 4 fields, an extra field was counted in piece 3 (total area of approximately 8.3 mm^2^).

### Whole cell lung homogenate

Rat lung tissue (0.1 g) were homogenized in 1 ml of ice-cold lysis buffer containing 1% Nonident 40 (NP-40), 0.1% SDS, 0.01 M deoxycholic acid and a complete protease cocktail inhibitor with EDTA (Roche, East Sussex, UK) and incubated on ice for 45 min. The samples were then centrifuged at 13,000 rpm for 25 min at 4°C and the supernatant aliquoted and stored at -80°C.

### Western Blotting

Lung tissue homogenate samples were separated on SDS polyacrylamide gel. Separated proteins were electroblotted onto nitrocellulose membranes (Schleicher and Schuell, Dassel, Germany) and blocked for 1 hr at room temperature with 5% nonfat dry milk. The membranes were incubated with anti-VEGFR2 (Santa Cruz, Santa Cruz, CA, USA), PI-3K (Upstate, Milton Keynes, UK), anti-Akt, anti-phosphoacetylated-Akt, anti-Bad, anti-phosphoacetylated Bad (Ser136), anti-Bcl-2, anti-Bcl-xl, anti-phosphorylated eNOS (Ser1177), anti-eNOS (Cell Signaling) and anti-β-actin (Santa Cruz Biotechnology).

### Immunoprecipitation

Lung homogenate (200 μg of protein) in a final volume of 100 μl lysis buffer was pre-cleared with Protein-A-agarose beads (Calbiochem, Merck Biosciences, Nottingham, UK) for 30 minutes at 4°C with constant agitation. The samples were then incubated with the antibody for 1 hr at 4°C with constant agitation. Protein-A-agarose beads were then added and left at 4°C overnight with constant agitation. The samples were then centrifuged, the supernatant discarded and the beads were washed in lysis buffer and heated with sample loading buffer. The samples were then run on Western blots using SDS-PAGE. Blots were probed with anti-PI-3K (Upstate) and anti-Bad (Cell Signaling) and stripped, reprobed with anti-Bcl-xl (Cell Signaling) or anti-VEGFR2 (Santa Cruz) as loading controls.

### Protein Assay

Protein level was measured with a bicinchoninic acid kit (Bio-Rad Laboratories Inc., Hercules, California, USA). Protein standards were obtained by dilution of a stock solution of BSA. Linear regression was used to determine the actual protein concentration of the samples.

### RNA isolation and reverse-transcriptase PCR

Lung tissue (0.1 g) was homogenized in 1 ml of Trizol (Invitrogen Life Technologies, Paisley, UK) and left at room temperature for 15 minutes. RNA was extracted according the manufacturer's instructions. The RNA was the aliquoted and stored at -80°C until further use. RNA was quantified by a spectrophotometer at 260 nm. Protein contamination was estimated at 280 nm and a ratio of < 1.6 was accepted. RNA (2 μg) was reverse transcribed in to cDNA using oligo-dT MMLV-Reverse Transcriptase in a 20 μl final volume. RT-PCR was then preformed using 5 μg of cDNA (primers see below) using 1× PCR buffer (50 mM KCl, 10 mM Tris-HCl pH 9.0, 1.5 mM MgCl_2 _and 0.1% Triton X-100), 400 μM dNTP, 1 mM MgCl_2_, 50 pM of each 5' and 3' primer and 2 U of *Taq *DNA polymerase (Promega). RT-PCR amplification was carried out using rat Bcl-2 up 5'-CCG GGA GAT CGT GAT GAA GTA-3'; Bcl-2 rev 5'-CAT ATT TGT TTG GGG CAT GTC T-3' and rat GAPDH).(36) as a loading control using previously described RT-PCR parameters [bcl2: 94°C for 45 sec; 58°C for 45 sec; 72°C for 60 sec for 25 cycles and then final 72°C for 10 min (product size 508 bp) and GAPDH: 95°C for 30 sec; 60°C for 60 sec; 60°C for 2 mins for 25 cycles and then 68°C for 7 mins (product size 520 bp)] [29]. RT-PCR reactions were carried out using a thermocycler and the PCR products were electorophoresed on a 1.5% agarose gel containing ethidium bromide (EtBr). The bands were visualised and quantified by densitometry using a UV Grab-IT software package.

### Statistical analysis

All the data are expressed as Mean ± SEM. Statistical analysis of significance was preformed using Minitab software. The data were normally distributed and values obtained in the different groups of rats were compared using one-way analysis of variance (ANOVA) using *Tukey's post-hoc test*. Statistical significance was considered at *P *< 0.05, *P *< 0.01 and *P *< 0.001 levels.

## Results

### Cigarette smoke regulates VEGFR-2-PI3K interaction in rat lung

**Figure 1:**
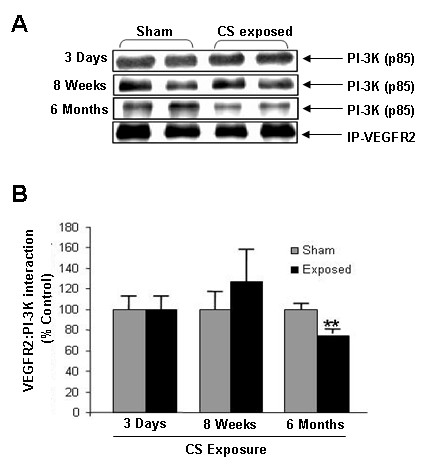
**VEGFR2-PI-3K association in rat lungs exposed to CS**. (**A**) A representative immunoblot picture of immunoprecipitated VEGFR2 probed for the p85 catalytic subunit of PI-3K after 3 days, 8 weeks and 6 months of CS exposure in rat lungs. The interaction of the p85 subunit of PI-3K was unaltered after 3 days and 8 weeks of CS exposure but was significantly increased after 6 months of CS exposure compared to sham-exposed animals (n = 6). (**B**) Histograms represent the Mean ± SE of percentage of VEGFR2/PI-3K association. ** *p *< 0.01 compared to sham-exposed animals.

Our previous studies have shown that CS reduced VEGFR2 phosphorylation and its total levels in mouse and rat lungs [28,29]. One of the key initiators of VEGFR2-mediated endothelial survival signaling is PI-3K. It is known that PI-3K interacts with VEGFR2 directly by the p85 catalytic subunit [11]. We therefore investigated the effect of CS on VEGFR2-PI-3K interaction in rat lungs (Fig. 1). No significant alteration was observed on PI-3K (p85 subunit) interaction with VEGFR2 after acute or sub-chronic CS exposure compared to sham-exposed animals. However, significant (*P *< 0.01) reduction was observed after 6 months of CS exposure suggesting that chronic CS exposure reduced VEGFR2-PI-3K interaction in rat lungs.

### Cigarette smoke reduced Akt phosphorylation

**Figure 2:**
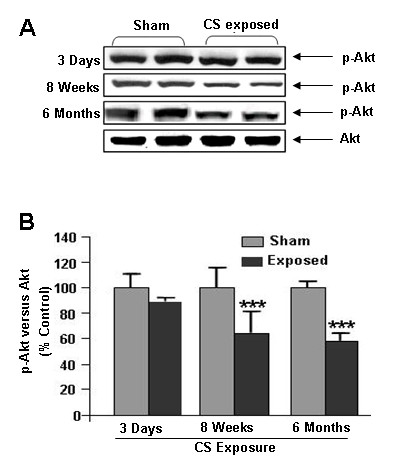
**Effect of CS on Akt phosphoryaltion**. (**A**) A representative immunoblot picture of Akt phosphorylation after 3 days, 8 weeks and 6 months of CS exposure in rat lungs. Akt phosphorylation was significantly reduced both at 8 weeks and 6 months, but not after 3 days of CS exposure, compared to sham-exposed animals (n = 6). (**B**) Histograms represent the Mean ± SE of percentage of Akt phosphorylation. *** *p *< 0.001 compared to sham-exposed animals.

Previous study have shown that activation of Akt is pivotal in VEGF/VEGFR2-mediated endothelial cell survival [10]. We assessed the effect of CS on Akt phosphorylation by immunoblotting in rat lung. We found that Akt phosphorylation was significantly (*P *< 0.001) reduced after sub-chronic and chronic CS exposure compared to sham exposed animals (Fig. 2). However, CS exposure did not alter the total Akt level in acute, sub-chronic and chronic exposure time points. This data suggested that sub-chronic and chronic CS exposure impaired Akt survival signaling but not with acute CS exposure in rat lungs.

### Chronic CS exposure led to decreased Bad phosphorylation

**Figure 3:**
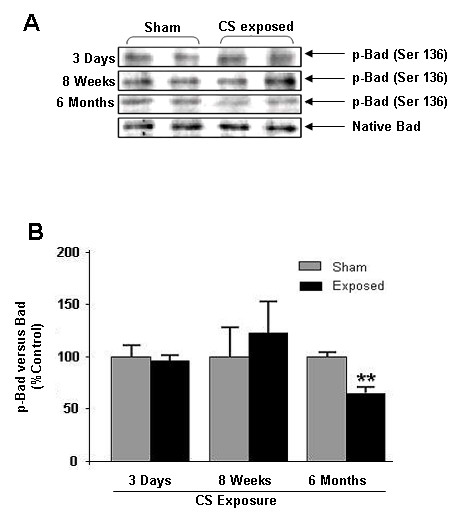
**Effect of CS on Bad phosphorylation**. (**A**) A representative immunoblot picture of Bad phosphorylation (Ser136) after 3 days, 8 weeks and 6 months of CS exposure in rat lung. Bad phosphorylation (Ser136) was unaltered after 3 days and 8 weeks but significantly decreased after 6 months of CS exposure compared to sham-exposed animals (n = 6). (**B**) Histograms represent the Mean ± SE of percentage of phosphorylated Bad levels. ** *p *< 0.01 compared sham-exposed animals.

Bad can inhibit anti-apoptotic signals of Bcl-2 and Bcl-xl, resulting in apoptotic cell death. Phosphorylation of Bad leads its inactivation and inhibition of Bad binding to Bcl-2 and Bcl-xl [22]. We therefore determined the effect of CS on Bad phosphorylation by immunoblotting. Phosphorylation of Bad (Ser136) was significantly (*P *< 0.01) reduced in chronic CS exposure compared to sham exposed groups (Fig. 3). However, acute and sub chronic CS exposures did not show any effect on Bad phosphorylation. The alterations of Bad phosphorylation in chronic CS exposure was correlated with decreased phosphorylation of Akt in rat lungs suggesting that CS exposure alters Akt-mediated survival signal by modulation on Bad (Ser136) phosphorylation.

### Cigarette smoke increased the Bcl-xl-Bad association

**Figure 4:**
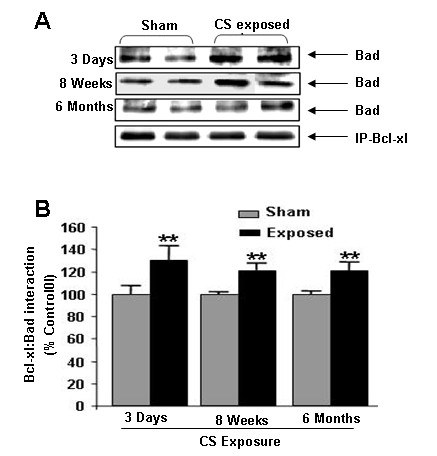
**Bad-Bcl-xl interaction in CS exposed rat lungs**. (**A**) A representative immunoblot picture of immunoprecipitated Bcl-xl probed for Bad after 3 days, 8 weeks and 6 months of CS exposed rat lungs. Bad interaction with Bcl-xl was significantly increased at 3 days, 8 weeks and 6 months CS exposure compared to sham-exposed animals (n = 6). (**B**) Histograms represent the Mean ± SE of percentage of Bad interaction with Bcl-xl. ** *p *< 0.01 compared to sham-exposed animals.

It has been shown that interaction of Bad with Bcl-xl inhibit its anti-apoptotic effect and activates apoptotic events [20]. Hence the interaction of Bad with Bcl-xl was assessed by immunoprecipitation and immunoblotting. We found that acute and chronic CS exposures significantly (*P *< 0.01) increased the Bad/Bcl-xl binding compared with sham exposed animals (Fig. 4). These data indicate that increased association of Bad/Bcl-xl may lead to increased apoptotic cell death in rat lungs.

### Cigarette smoke had no affect on Bcl-2 mRNA expression

**Figure 5:**
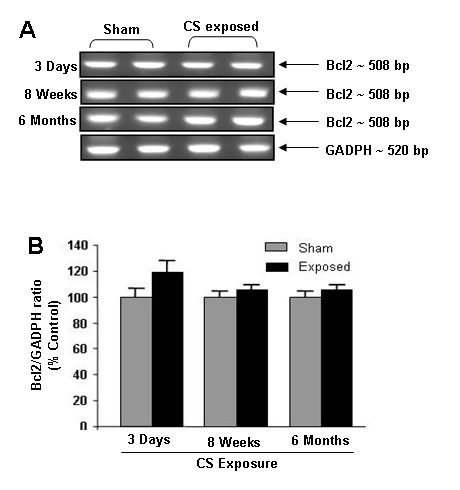
**Bcl-2 mRNA levels in CS exposed rat lung**. (**A**) A representative RT-PCR picture of Bcl-2 mRNA levels after 3 days, 8 weeks and 6 months of CS exposure. Bcl-2 mRNA expression remains unaltered at all time point compared to sham-exposed animals (n = 6). (**B**) Histograms represent the Mean ± SE of percentage of Bcl-2 mRNA expression levels.

It has been shown that VEGF/VEGFR2-mediated survival signal may be mediated through sustained upregulation of Bcl2 expression [18]. CS had no effect on expression of Bcl-2 mRNA as measured by RT-PCR in both acute and chronic time points compared with sham operated animals (Fig. 5). This data suggests that CS does not affect Bcl-2 mRNA expression in response to either acute or chronic exposures in rat lungs.

### Cigarette smoke reduced the eNOS level and its phosphorylation

**Figure 6:**
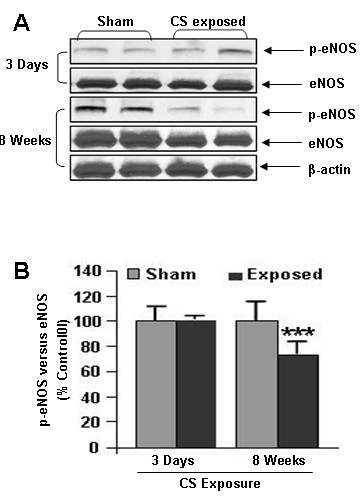
**Phosphorylated and total eNOS levels in CS exposed rat lung**. (**A**) A representative immunoblot picture of phosphorylated and total eNOS after 3 days and 8 weeks of CS exposure in rat lungs. Phophorylated and total eNOS levels were significantly reduced in 8 weeks, but not after 3 days of CS exposure compared to sham-exposed animals (n = 6). (**B**) Histograms represent the Mean ± SE of percentage of eNOS phosphorylation. *** *p *< 0.001 compared to sham-exposed animals.

VEGF-induced VEGFR2 phosphorylation and downstream signaling leading to activation of eNOS, a key enzyme linked to endothelial survival and function. Therefore, the effect of CS on phosphorylated and total eNOS levels was assessed by immunoblotting. CS significantly (*P *< 0.001) reduced the level of phophorylated and total eNOS compared to sham-exposed rat lungs after sub-chronic exposure without any significant change after acute exposure (Fig. 6). We expect a similar reduction in total and phosphorylation eNOS after chronic CS exposure compared to sham-exposed rat lungs. These data suggested that chronic CS exposure impairs the activation of eNOS in rat lungs which may have further implication on decreased NO production and endothelial dysfunction.

### Cigarette smoke exposure had no effect on activation of caspase 3 or lung cell death

**Figure 7:**
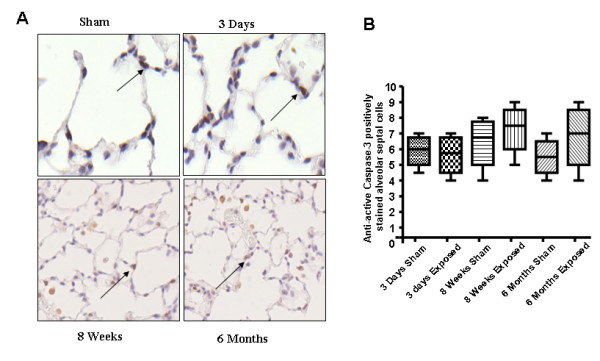
**Effect of CS smoke on active caspase 3 activation in rat lungs**. (**A**) Representative pictures show the IHC staining of active caspase 3 at 3 days, 8 weeks and 6 months CS smoke-exposed rat lungs. Arrows represent cells positively stained for active caspase 3. (**B**) Graph representing positive cell counts in rat lungs. There was no difference in the number of positively stained cells between sham-exposed and CS-exposed animals after either 3 days, 8 weeks or 6 months of CS exposure in rat lungs (n = 6).

Increased endothelial cell death was observed in emphysematous lungs of smokers indicate that apoptotic cell death may play a role in pathogenesis of COPD [4]. To investigate the effect of CS on lung cell death, expression of active caspase 3 was assessed by immunohistochemmistry. There was no difference in active caspase 3 expression between CS exposed and sham-exposed animals after either acute or chronic exposures (Fig. 7). These data suggested that CS exposure was not associated with lung cell death.

## Discussion

VEGFR2-mediated Akt survival signaling has been shown to be critical in endothelial cell survival [10]. Previous studies have shown that emphysema patients have decreased VEGF and VEGFR2 expression along with increased endothelial cell death [4,29]. Moreover, inhibition of VEGFR2 has also showed increased lung endothelial cell death in rats [12]) VEGFR2 activates Akt by interacting with PI-3K through its p85 subunits [10,11]. In our data we observed significant alteration in VEGFR2/PI-3K interaction after chronic CS exposure in rat lungs. This supports our concept that chronic CS exposure in rat lungs reduces the interaction of both PI-3K and VEGFR2 thus further leads to alteration of Akt-mediated downstream survival signaling.

Our previous studies showed that CS significantly decreases the VEGFR2 and Akt levels in mouse and rat lungs [28,29], and this is likely to be linked to VEGFR2-mediated survival signaling. In present study, we show that Akt phosphorylation was significantly reduced after sub-chronic and chronic CS exposures compared to sham-exposed animals, which was directly correlated with decreased PI-3K/VEGFR2 interaction on CS exposed animals. This altered VEGFR2/PI-3K association and impaired Akt phosphorylation may further leads to modifications on its downstream targets via Bad phosphoryation. The reason for CS-mediated reduction of VEGFR2 is not known but our recent study suggested that VEGFR2 is post-translationally modified by ROS/RNS present or derived by CS [30].

The pro-apoptotic Bad is the primary target of Akt and Akt phosphorylates Bad and rendering it inactive for apoptotic signal [20]. In this study, we show that after 6 months of CS exposure, there was a significant decrease in Bad phosphoryation (Ser136) in lungs as compared to sham-exposed animals. These data are consistent with the reduction of Akt phosphorylation seen in the CS exposed animals, and indicates that decreased phosphorylation of Bad may further increase its association with Bcl-xl. The lack of any change seen after acute and sub-chronic CS exposure may be due to a cross-talk with other receptors and signaling pathways, compensating for any decrease in Akt activation or the decrease seen in Akt activation was not sufficient to impact on Bad Ser136 phosphorylation levels. However, further studies are required to clarify the role played by Bad phosphorylation in response to CS exposure in lung cell death.

Bcl-xl is an anti-apoptotic protein that promotes cell survival by inhibiting caspase-mediated apoptotic cell death [20]. Heterodimerization of Bad with Bcl-xl prevents anti-apoptotic effect of Bcl-xl [31]. Bcl-xl can also binds directly to the outer membrane of the mitochondria, forming a pore to allow anionic metabolite exchange across the membrane and promoting cell survival during apoptotic signaling [32]. Native Bad can bind to Bcl-xl, displacing Bax and preventing the Bcl-xl binding to the mitochondria membrane [20]). Hence, we studied the Bcl-xl/Bad interactions in acute, sub-chronic and chronic CS exposed animals. We found that Bcl-xl/Bad interaction was significantly elevated after acute and chronic CS exposures in rat lungs. However, there was no alteration in Bad phosphorylation (Ser136) after acute and sub-chronic CS exposures indicating that this elevated Bad-Bcl-xl interaction may be independent of Bad phosphorylation at this site. Bad is also phosphorylated by protein kinase C at Ser155. Phosphorylation of Bad at Ser155 is also thought to play an important role in prevention of dimerization of Bad to Bcl-xl due to its position on the BH3 domain [33]. Therefore, further studies are required to assess the role of phosphorylated and total levels Bad in apoptotic-mediated cell death.

VEGF-mediated VEGFR2 phosphorylation and its downstream signaling via Akt induced the activation of eNOS in endothelium (24). It has been shown that eNOS inhibits apoptosis and increase cell survival through Bcl-2-dependent pathway [27]. Since eNOS is also an essential mediator of VEGFR2-mediated endothelial survival, we determined whether CS exposure had any effect on phosphorylated and total eNOS levels. Our data showed that chronic CS exposure decreased the phosphorylated and total eNOS levels in rat lungs. These data are in agreement with reduction in Akt phosphorylation in response to CS. Recently, we have shown that CS impairs the VEGF induced VEGFR2-mediated eNOS phosphorylation levels in human microvascular endothelial cells [30]. Upon activation by its upstream kinases eNOS release NO in endothelial cells, which is known to mediates cell survival and resistance to apoptosis [25]. Hence this data further support our concept that CS decreases VEGFR2-mediated downstream signaling thus leading to diminished NO production and cell survival.

**Figure 8:**
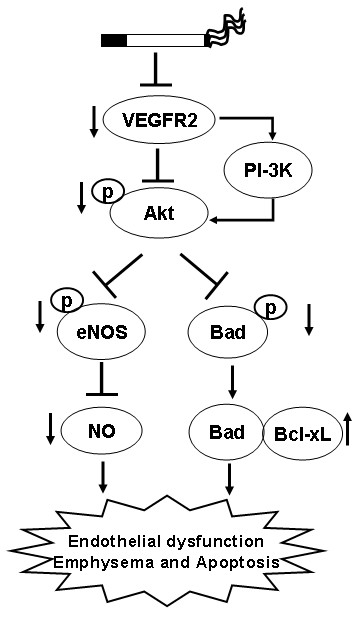
**Hypothesized mechanism of CS-impaired VEGFR2-mediated survival signaling**. CS decreases VEGFR2 levels thereby alters the survival signaling via PI-3K. Downregulation of VEGFR2-PI-3 K-Akt pathways will lead to reduction of eNOS and NO bioavailability as well as reduced survival signaling via increasing the Bad-Bcl-xL interaction.

Caspases, in particular caspase 3, are important marker of cell undergoing apoptotic cell death. CS had no effect on the level of active caspase 3 in lung cells. This data corroborates with previous studies demonstrating that lung cell death events were not significant between smokers and non-smokers though CS causes destruction of alveolar wall and reduction in vascular density only in emphysematous lungs [4]. We have recently reported that emphysema-like changes were observed after 8 months of CS exposure in rat model [34]. It is possible that 6 months of CS exposure may explain the noticeable changes and different events of apoptotic cell death. Taken together, our data indicate that CS exposure alters VEGFR2-mediated survival signaling in rat lungs. However, despite the reduced VEGFR2-PI3-K association, Akt activation, Bad phosphorylation and increased Bad/Bcl-xl interaction, the studied time points were unable to explain the CS induced global lung cell death. It is important to note that impaired VEGFR2-mediated survival signaling pathway may have further implication on CS-mediated endothelial cell apoptosis. Further investigations are required to substantiate the VEGFR2-mediated cell survival signaling mechanism in CS-induced emphysematous lungs. In conclusion, CS downregulates VEGFR2-mediated cell survival signaling pathways in rat lungs *in vivo *(Fig 8), however, these alterations were unable to induce apoptotic-mediated cell death. These findings suggest that in human lungs, as it is possible that CS exposure does not cause lung cell apoptosis unless lungs have undergone airspace enlargement. As emphysematous lungs, but not smokers' lungs, display reduced endothelial cell survival and vascular regression; the lungs of the rat exposed to chronic cigarette smoke may be representative of smokers whereas emphysema is known to be associated with lung cell apoptosis (airspace enlargement).

## Abbreviations

CS: cigarette smoke; COPD: chronic obstructive pulmonary disease; eNOS: endothelial nitric oxide synthase; VEGF: vascular endothelial growth factor; VEGFR2: VEGF receptor 2.

## Competing interests

The authors declare that they have no competing interests.

## Authors' contributions

JAM, IE and GA contributed in the study design and planning, and performed the experiments. JAM, IE and GA performed immunoassays, immunoblottings and cell counts. IE and GA performed chronic smoke inhalation experiments. JAM, IE and GA performed the statistical analysis. JAM wrote the first draft of the manuscript. IE and GA revised the subsequent drafts. CSS performed the cigarette smoke *in vivo *inhalation experiments along with some studies by IE and GA. WM, PAK and IR supervised the study and contributed in data discussions and correcting the drafts. IR conceived the study, contributed in the study design, planning and revised the manuscript as well as handled the publication process with PAK. CSS and WM participated in designing the experiments and coordinated in completing the study. All authors read and approved the final manuscript.
